# Application of a *Neisseria meningitidis* antigen microarray to identify candidate vaccine proteins from a human Phase I clinical trial

**DOI:** 10.1016/j.vaccine.2022.05.032

**Published:** 2022-05-21

**Authors:** Chun-Mien Chang, Amaka M. Awanye, Leanne Marsay, Christina Dold, Andrew J. Pollard, Christine S. Rollier, Ian M. Feavers, Martin C.J. Maiden, Jeremy P. Derrick

**Affiliations:** a Lydia Becker Institute of Immunology and Inflammation, School of Biological Sciences, Faculty of Biology, Medicine and Health, Manchester Academic Health Science Centre, University of Manchester, Manchester M13 9PL, UK; b Oxford Vaccine Group, Department of Paediatrics, University of Oxford, and the NIHR Oxford Biomedical Research Centre, Oxford OX3 7LE, UK; c School of Biosciences and Medicine, University of Surrey, Guildford, UK; d National Institute for Biological Standards and Control, Blanche Lane, South Mimms, Potters Bar, Hertfordshire, UK; e Department of Zoology, University of Oxford, 11a Mansfield Road, Oxford OX1 3SZ, UK

**Keywords:** *Neisseria meningitidis*, Vaccine, microarray, Antigen, Serum bactericidal assay

## Abstract

Meningococcal meningitis is a rare but serious condition affecting mainly children and young adults. Outer membrane vesicles (OMV) from *Neisseria meningitidis* have been used successfully as vaccines against the disease, although they only provide protection against a limited number of the many existing variants. There have been many attempts to identify suitable protein antigens for use in defined vaccines that provide broad protection against the disease, such as that leading to the development of the four component 4CMenB vaccine. We previously reported the use of a protein antigen microarray to screen for IgG antibodies in sera derived from human recipients of an OMV-based vaccine, as part of a Phase I clinical trial. Here, we show that computational methods can be used to cluster antigens that elicit similar responses in the same individuals. Fitting of IgG antibody binding data to 4,005 linear regressions identified pairs of antigens that exhibited significant correlations. Some were from the same antigens in different quaternary states, whilst others might be correlated for functional or immunological reasons. We also conducted statistical analyses to examine correlations between individual serum bactericidal antibody (SBA) titres and IgG reactivity against specific antigens. Both Kendall’s tau and Spearman’s rank correlation coefficient statistics identified specific antigens that correlated with log(SBA) titre in five different isolates. The principal antigens identified were PorA and PorB, RmpM, OpcA, and the type IV pilus assembly secretin, PilQ. Other minor antigens identified included a lipoprotein, two proteins from the BAM complex and the efflux channel MtrE. Our results suggest that consideration of the entire antigen composition, and allowance for potential interaction between antigens, could be valuable in designing future meningococcal vaccines. Such an approach has the advantages that it uses data derived from human, rather than animal, immunization and that it avoids the need to screen individual antigens.

Invasive Meningococcal Disease (IMD), principally meningitis and septicaemia, is predominantly caused by encapsulated variants of the Gram-negative diplococcus *Neisseria meningitidis*. The meningococcus is a highly diverse organism, which is categorised into variants on the basis of both genetic and antigenic properties. Meningogoccal serogroups, are based on the capsular polysaccharide (CPS) with most cases of disease originating from A, B, C, W, X, and Y [1]. Vaccines containing conjugated serogroup A, C, W, and Y polysaccharides have been highly effective [2] but vaccination with serogroup B has not been exploited, due to safety concerns as this polysaccharide has structural similarities to polysialic acid groups found in human foetal brain tissue [3]. One alternative approach is vaccination with outer membrane vesicles (OMVs), which have been used effectively in response to outbreaks of, and endemic, serogroup B IMD [4–7]. OMV vaccine preparations are derived by detergent extraction from live growth cultures and are a complex mixture of outer membrane proteins (OMPs), capsule, lipopolysaccharide (LPS), and other components.

Meningococcal antigenic variability is a major drawback with OMV-based vaccines. The major OMV antigen is PorA, a pore-forming protein or porin, which is highly abundant in the outer membrane and is responsible for the passage of low molecular weight solutes into the periplasm. PorA is, however, highly variable among meningococci, particularly within its external loop regions [8]. Protection induced by OMV vaccination is therefore limited to those meningococci expressing the same or immunologically cross-reactive PorA variants. This led to the investigation of other OMP antigens that are less subject to sequence variation but which can contribute to protective immunity. Systematic approaches have used bioinformatics to identify candidate outer membrane antigens from genome sequence information followed by immunological assessment in mice [9]. This ‘Reverse Vaccinology’ [10] approach was used as a basis for the development of the multicomponent vaccine 4CMenB, (Bexsero^®^), which comprises three recombinant antigens (two as fusion proteins) and an OMV preparation [11]. 4CMenB has been shown to be effective in post implementation analysis of the incidence of serogroup B disease in England, which estimated an effectiveness of 59% [7], similar to the efficacy of 57.2% reported in 1991 from a phase III trial of an OMV vaccine generated against an outbreak strain in Norway in 1991 [12].

Important questions remain concerning the role of other antigens present in OMV-based vaccines, including 4CMenB. Clinical trial data showed that inclusion of an OMV preparation improved immunogenicity over preparations containing the recombinant proteins alone [13,14]. The precise OMV antigens that contribute to immunogenicity and protection remain unclear, as is the quantification of their relative contributions. Proteomic analyses of OMV content have identified well over 100 different proteins, including many that adopt β-barrel transmembrane folds [15–17]. This group of integral membrane proteins are challenging for vaccine development, as they can be more difficult to express and purify than soluble proteins. We sought to investigate this question using a dedicated antigen microarray, which incorporated the most abundant *N. meningitidis* integral outer membrane proteins [18]. The microarray was screened with sera derived from participants in a Phase I clinical trial of an OMV vaccine, MenPF-1. MenPF-1 was an OMV vaccine derived from the meningococcus used for the generation of the Norwegian vaccine that had been modified to constitutively express a variant of the iron transporter FetA [19], another variable OMP.

The results highlighted the importance of the porin proteins PorA and PorB, as well as the adhesin OpcA and the pilus biogenesis protein PilQ, as antigens that generate strong IgG responses. However, the widely accepted correlate of protection against IMD is the ability of antibody to activate complement-directed killing, as measured by the serum bactericidal antibody (SBA) titre. SBA activity originates from a subset of IgG antibodies that direct complement-dependent killing. This observation suggests that data on IgG recognition of individual antigens could be used to examine correlation with SBA titres. This approach has been used recently by Bartolini et al., who employed a protein microarray to record the antibody responses to peptide fragments derived from three principal 4CMenB recombinant vaccine antigens: (i) factor H binding protein (fHbp); (ii) heparin binding protein (NHBA); and (iii) Neisserial adhesin A (NadA) [20]. These antigen ‘fingerprints’ were then correlated with SBA responses in vaccinated individuals, providing insight into the relationship between portions of the sequence from the three recombinant antigens and SBA titre. Here, we show how data from an antigen microarray screened with sera from individuals vaccinated with an OMV-based vaccine can be used to identify correlations between IgG antibody responses against specific antigens [18]. We further demonstrate that statistical correlation methods can be applied to identify antigens that contribute to bactericidal responses induced by the OMV vaccine. The results are summarised as a linear regression model that uses IgG responses to a select group of antigens to predict log (SBA), showing how the key component antigens within an OMV vaccine can be identified and their contributions to SBA quantified.

## 1 Experimental

### 1.1 Microarray data

Microarray data were obtained from a previously published study [18]. Briefly, to construct the microarray, open reading frames for recombinant proteins were designed and cloned into pOPINF and pOPINE expression vectors by the Oxford Protein Production Facility. Expression was carried out in BL21 (DE3) E. coli cells (New England Biolabs); cultured cells were grown in Terrific Broth at 16 °C, induced by addition of isopropyl-β-1-D-thiogalacto pyranoside (IPTG; Sigma-Aldrich) to a final concentration of 0.5 mM and grown for 12–14 h before harvesting. Insoluble polypeptides, which were generally from membrane-spanning proteins, were expressed into inclusion bodies, recovered by solubilisation into chaotrope and refolded by dilution, as described previously [21]. All target proteins were purified by metal chelate followed by size exclusion chromatography (further details are given in Awanye *et al*. [18]). All purified OMPs were concentrated to 5–10 mg/ml, protein concentration estimated from absorbance at 280 nm and stored at −80 °C prior to microarray printing. Microarray development and data processing was carried out as described previously [18].

### 1.2 MenPF-1 OMV vaccine and Phase I clinical trial

The MenPF-1 vaccine was an OMV vaccine produced from genetically modified *N. meningitidis* isolate H44/76, which had been manipulated to constitutively express the iron transporter FetA [22], formulated with aluminium hydroxide, sucrose and water [18]. The data used here were derived from 24 volunteers in receipt of 50 μg of MenPF-1. Three doses of vaccine were administered by intramuscular injection 8 weeks apart and serum samples were collected 4 weeks after each dose. Samples used for this study were pre-vaccination, and 12- and 20-weeks post-vaccination. Ethical approval for the study was granted by NRES Oxford A (12/SC/0023) and the trial was registered with clinicaltrials.gov and EudraCT (NCT01640652 and 2012–001046-17 respectively). The trial was conducted in accordance with the principles of the Declaration of Helsinki (2008) and the International Conference on Harmonization (ICH) Good Clinical Practice standards.

### 1.3 Serum bactericidal assays

Serum bactericidal titres were measured using 25% vol/vol human complement without intrinsic bactericidal activity, obtained from healthy donors. The clinical study OVG 2009/07, was approved by the research ethics committee South West 4, reference 10/H0102/23, and published previously [19] as % of participants with titers ≥ 1:4. Here the individual SBA titres are reported for each dose and each time point tested.

### 1.4 Statistical analysis

Spearman rank correlation coefficient (rho) was calculated in R using the package cor and plotted using corrplot. t-distributed stochastic neighbour embedding (tSNE) calculations were carried out using the package Rtsne (https://github.com/jkrijthe/Rtsne). Hierarchical cluster analysis was implemented using pvclust, as described by Suzuki and Shimodaira [23]. Statistical analysis for the data presented in Tables 1 and 2 was conducted using SPSS version 25 (IBM). Bayes factor analysis was conducted using the BayesFactor package (version 0.9.2+) [24].

## 2 Results

**Fig. 1:**
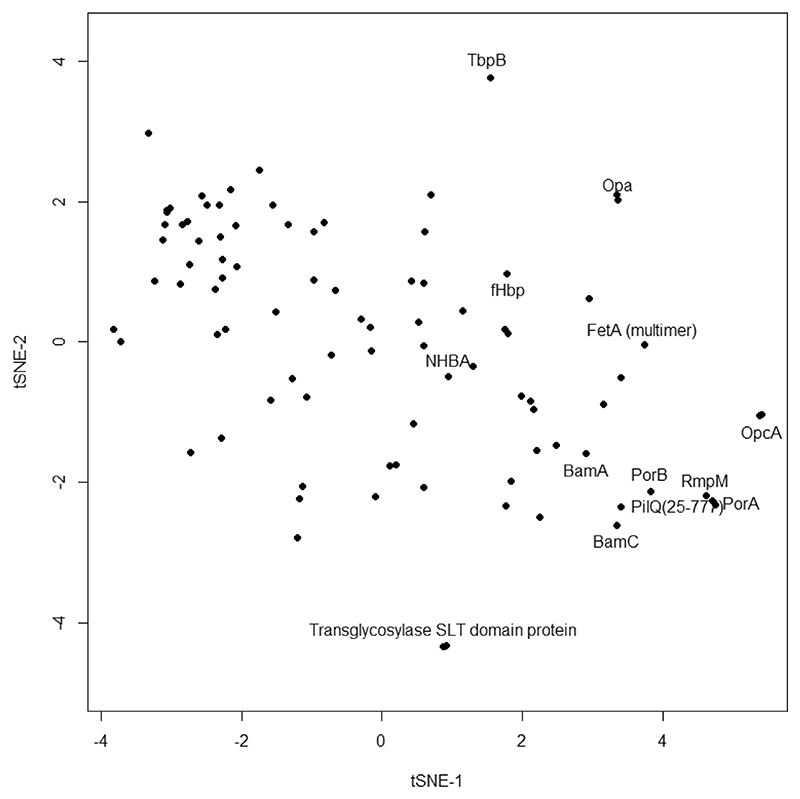
tSNE separation of IgG responses to specific antigens following vaccination with MenPF.. Data from 12 and 20 week measurements were combined. Perplexity used was 30. Selected antigens are labelled.

Initial analysis of antigen microarray data from the MenPF-1 trial employed a paired *t*-test that analysed increases in IgG binding to antigen after vaccination relative to baseline (pre-vaccination sera) [18]. This approach identified major responding antigens but potentially missed other signals, particularly relationships in responses among antigens. An alternative approach considered each antigen as a vector with multiple dimensions (IgG binding values), at the 12- or 20-week time points. The tSNE, t-distributed stochastic neighbor embedding [25], method collapses such a matrix down to two dimensions (Fig. 1). Antigens that elicit similar responses within vaccine recipients are grouped together on the plot i.e. if individuals who respond well to antigen A are also more likely to respond to antigen B, then both antigens will be closely located. In this case, the antigens inducing the strongest responses are in the bottom right, including the major porin PorA. Other closely associated antigens are the other porin PorB, the reduction modifiable protein RmpM, the PilQ secretin, and BamA/BamC from the BAM complex. Grouping of these antigens suggests that vaccinated individuals who respond well to one of them also responded well to the others. It is noteworthy that some of the other antigens within the plot lie to the extremes: the transglycosylase SLT domain protein, or an Opa protein, for example. The fact that these antigens are located more remotely from the PorA grouping suggests that their pattern of responses in vaccinated individuals differed.

**Fig. 2:**
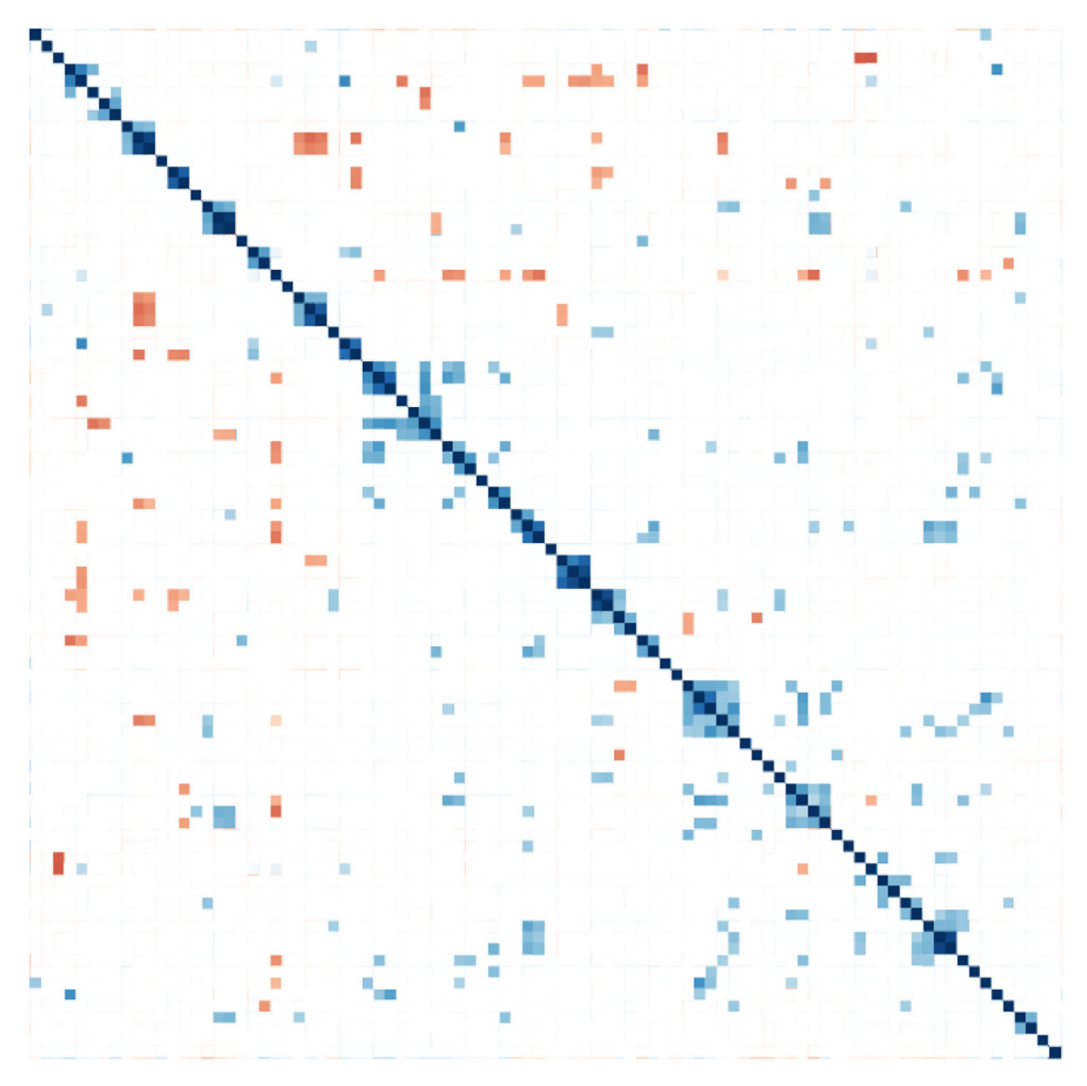
Pairwise Spearman rank correlations between antigens. Spearman rank correlations were calculated using a p value cutoff value of 0.1%. Points are coloured by Spearman rho on a scale from 1.0 (blue) to −1.0 (red). Ward clustering was imposed to group correlated antigens. Analysis used data from 0, 12 and 20 week time points. (For interpretation of the references to colour in this figure legend, the reader is referred to the web version of this article.)

It is a common observation that IgG responses to particular antigens vary within a vaccinated population e.g. the IgG responses to the PorA porin (Fig. 1). As anticipated, most responses are low at pre-immunization: responses at the 12- and 20-week points are generally higher but vary over an order of magnitude. To examine whether variability in responses to a particular antigen, such as PorA, correlated with relatively higher or lower responses to any other antigen, all IgG binding values were analysed at the 12- and 20-week time points, with normalized and Spearman rank correlations determined for all combinations (Fig. 2). The relationships were analysed by hierarchical clustering, which enabled grouping of antigens by similarity of response (Fig. S2). The results indicated that there were groups of antigens that exhibited correlated immune responses, either positively or negatively. This could be due to a cross-reaction of antibody between antigens that contain similar epitopes. Alternatively, the antigens might be physically associated in some way, e.g. hierarchical clustering places PorB and RmpM close together. Although there is no significant sequence homology between these proteins, RmpM binds to PorB and stabilizes the trimer [26]. It is also possible that given individuals are pre-disposed to stronger or weaker humoral responses to certain groups of antigens.

**Fig. 3:**
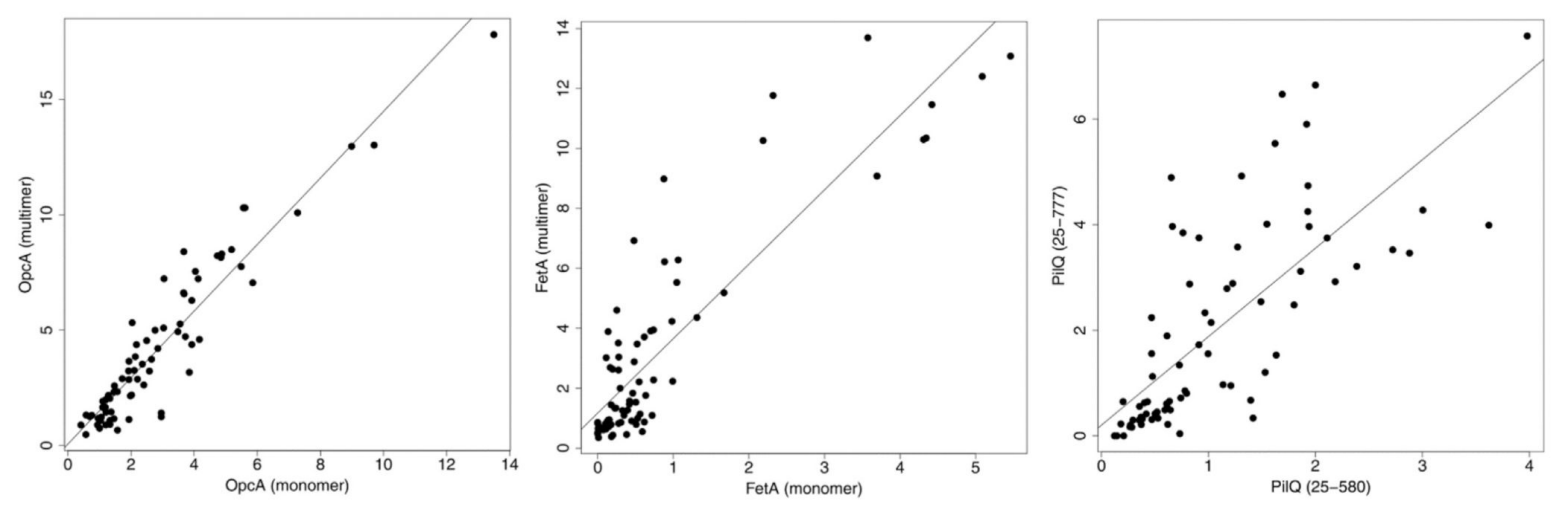
Examples of correlations between IgG responses to similar antigens. Correlations are shown after normalization using caret, implemented in R. F-statistics and p-values are for OpcA, 558.5 and < 2.2E-16, for FetA, 253.7 and < 2.2E-16, and for PilQ, 84.3 and 9.597E-14. Analysis used data from 0, 12 and 20 week time points.

To examine this phenomenon in more detail, linear regression analysis was performed on the IgG responses for each antigen-antigen pair. Computation of the F-statistic provided a metric that identified the most correlated antigen pairs. After removal of self-correlations, a total of 4,005 potential interactions were analysed. Of these, the highest 46, when ranked by F-statistic, are presented in Table S1. Some of the stronger correlations were between the same antigens in different oligomeric forms. It was often the case during purification of proteins for the microarray that particular antigens would elute from size fractionation by SEC as monomers and multimers. Under these circumstances, both peaks were collected and included separately on the microarray. An example of a strong correlation is shown for OpcA (Fig. 3, left panel); although crystallographic studies have shown that OpcA packs as a monomer [27], our earlier study of 2D crystalline arrays indicated oligomer formation [28]. Cross-reaction of IgG between both forms is anticipated, as many epitopes will be shared in both states. As a second example, with a weaker reacting antigen, there was a correlation with FetA (Fig. 3). Our previous crystallographic studies have shown that FetA forms both monomers and trimers in the crystalline state [29]. The final example is the secretin PilQ, which forms a transmembrane channel for the passage of type IV pili. Two forms of PilQ were included: one comprises just the domains predicted to lie in the periplasm (25–580) and the second includes the C-terminal transmembrane-spanning segment (25–777) [30]. It is reasonable to infer that the correlation arises from antibodies against the periplasmic domains which cross-react against both forms.

**Fig. 4:**
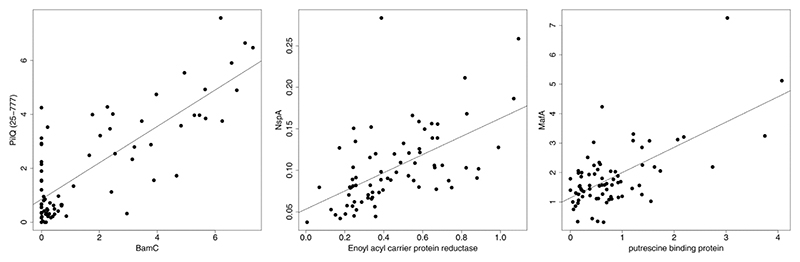
Examples of correlations between IgG responses to dissimilar antigens. Correlations are shown after normalization using caret, implemented in R. F-statistics and p-values are, for PilQ/BamC, 124.7 and < 2.2E-16, for NspA/Enoyl_acyl_carrier_protein_reductase, 33.64 and 1.642E-07, and for MafA/ putrescine binding protein, 51.88 and 4.672E-10. Analysis used data from 0, 12 and 20 week time points.

Of interest are interactions between different antigens, rather than the same antigen in different states: examples for three pairs of antigens are shown in Fig. 4. BamC is a component protein from the BAM complex, which is responsible for insertion of integral membrane proteins into the outer membrane [31]. It is likely that a secretin, such as PilQ, could interact with the BAM proteins during assembly; indeed, there is evidence that mutation to Omp85 (another BAM component) leads to mis-insertion of the secretin [32]. The precise function of NspA, an 8-stranded beta barrel integral OMP, is unclear, although the crystal structure showed evidence for a ligand binding site occupied by a detergent molecule [33]. The association with enoyl acyl carrier protein reductase is therefore intriguing. Other associations were harder to rationalise and may not have their explanations in any kind of functional association. Nevertheless, they merit further investigation and indicate that specific pairings of antigens within a vaccine formulation could influence antibody responses.

SBA titres were available for sera derived from each vaccine recipient at the 12- and 20-week time points. Statistical methods were employed to examine the correlation of each IgG antigen response with the logarithm of the SBA titre. This approach makes use of the fact that individual SBA titres against OMV antigens varied. An individual with a high SBA titre would be expected to show good antibody responses to key antigens that contribute to such a high SBA measurement. Separate correlation analyses were carried out on 5 meningococci: the original H44/76 wt, PorA- and PorA-FetA-mutants in the same H44/76 background, and two further variants, one of which was FetA-. Two separate statistical correlation studies were carried out: Kendall’s tau (Table 1) and Spearman’s Rank Correlation Coefficient (Table 2). Both forms of statistical analysis identify PorA as the antigen most prominently correlated with log(SBA titre). This observation is consistent with the fact that PorA is well established as the principal protective antigen within OMV-based vaccines. The Kendall’s tau or Spearman rho values for PorA provide a useful quantification for the relative contribution of antibodies against other antigens. So, the tau value for PorA of 0.53 against the WT strain compares with values ranging from 0.37 to 0.25 for other antigens (the equivalent Spearman’s rho is 0.73 for PorA compared to 0.52 through 0.34). As anticipated, the tau and rho values were much reduced or fell below the significance cutoff value in the PorA-strains. In the case of the PorA-FetA+), the PorA correlation values were reduced to similar values for some other antigens, such as PorB. This may be attributable to cross-reaction of anti-PorA antibodies against other antigens.

Comparison of linear regression models was used as a second approach to examine the contribution to the SBA titre made by antibodies to antigens other than PorA. An ANOVA analysis was carried out on a comparison between models which included either PorA antibody responses alone, or with PorA plus the six antigens that consistently recorded significant correlations. For SBAs obtained from all three strains that contained PorA, the model that included additional antigens provided a better fit to the data (Table 3). We also employed an alternative approach for comparison of multiple linear regression models using Bayesian factor analysis, as developed by Liang *et al*. [34]. Inclusion of RmpM and BamA, compared with PorA alone, gave Bayes factors of 8.67 ± 0.01% (wild type), 3.48 ± 0.01% (3208 FetA+) and 6.73 ± 0.01% (3043 FetA-). A value greater than 1.0 indicates additional benefit to inclusion of antigens other than PorA; this was consistently the case when incorporating the antigens identified in Tables 1 and 2 in different combinations. Overall, these analyses established that inclusion of the antibody responses to additional minor antigens was more predictive of SBA responses.

## 3 Discussion

Despite recent advances [7,10,11], there is much scope for the improvement of vaccines against serogroup B IMD. Development of an improved multi-component vaccine against *N. meningitidis* represents a complex challenge, however. Identification of the three main recombinant antigens in 4CMenB (fHbp; NHBA; and NadA) was achieved through screening of individual recombinant antigens [9,11]. The fHbp antigen was simultaneously discovered independently by an extensive conventional vaccinology study [35]. Here we have investigated an alternative approach, exploiting statistical correlations between IgG responses to specific antigens and SBA titre, a widely accepted correlate of protection. This method is based on the intrinsic variability in IgG responses and SBA titres within the vaccinated cohort. It is encouraging that significant statistical correlation values for IgG reactivity against specific antigens with SBA titre can be observed from a trial with only 24 participants (Tables 1 and 2).

Many of the outer membrane proteins identified by our statistical screen have been investigated as potential vaccine components previously. PorB forms the other major porin in the meningococcal outer membrane and has been advocated as a vaccine component [36]. Both PorA and PorB are bound to peptidoglycan within the periplasm by RmpM. RmpM is made up of a C-terminal globular domain, which binds the DAP moiety from peptidoglycan [26,37], and a flexible N-terminus which is thought to bind to the porin on the periplasmic side of the OM [36]. Given the abundance of PorA and PorB in the outer membrane, the strong response elicited by RmpM is not surprising, although it is puzzling that it is linked to SBA titre as the current model for its function indicates that it is not surface-exposed. It is also interesting to note that antibodies against the corresponding gonococcal protein block access to bactericidal antibodies against PorB [38].

OpcA, in both its monomeric and multimeric forms, features strongly in the Spearman and Kendall correlation analyses observed for PorA + meningococci tested although, interestingly, that is not the case for the two PorA deletion strains. OpcA is an integral outer membrane protein that forms a 10-stranded beta barrel structure with surface-exposed loop regions [27]. OpcA is known to bind to proteoglycan ligands and its specificity has been identified for sialic acid-containing ligands [39]. Bactericidal antibodies against OpcA have previously been observed in vaccinated individuals [40]. Expression of OpcA is subject to phase variation which implies that there is likely to be considerable strain to strain variability in SBA responses [41]. Opc is also absent from some lineages of *N. meningitidis*.

PilQ is a member of the secretin family, a diverse group of integral outer membrane proteins that form portals within the outer membrane for the secretion of pili and other substrates. Understanding of these complex proteins, which form ring-like structures in the outer membrane [28,42], has been accelerated in recent years due to structural analyses [43]. Secretins comprise multidomain structures, with a conserved C-terminal region that spans the outer membrane [30]. We previously identified two regions of PilQ, which both fall within the C-terminal region, and exhibit relatively high sequence variability. By analogy with studies on PorA and PorB, we interpret such sequence variability as a consequence of immune selection [44,45]. One region is a ‘cap’ structure, which lies outside the predicted membrane location. The second is part of the ‘periplasmic gate’, a structure found in other secretins that seems to play a role in regulating substrate passage through the channel in the outer membrane. We presume that the structure would need to move aside to allow passage of the pilus fibre and thus become surface-exposed in the complete assembled type IV pilus machinery *in vivo* [46]. Of note here is the observation that the longer PilQ variant, 25–777, is more closely associated with log(SBA) titre than the 25–580 form: the latter comprises just the periplasmic domains and not the cap or periplasmic gate structures.

It is noteworthy that the iron transporter FetA did not feature among the most strongly correlated antigens in Tables 1 and 2. In a recent study of the same Men-PF1 trial we have reported low FetA-specific memory B cell responses and anti-FetA IgG antibody levels which were only modestly raised after OMV vaccination [47]. It is likely that FetA-specific antibody responses in the microarray data were not sufficiently strong to exceed our correlation cutoff value (p < 0.01).

The correlation analysis identified two members of the BAM complex, BamA and BamC. The BAM assembly comprises five proteins that promote the assembly and insertion of integral outer membrane proteins [48]. BamA is an integral OMP itself and the crystal structure of *Neisseria gonorrhoeae* BamA exhibits several loop regions that could be recognized by external antibody [49]. BamC, by contrast, is not predicted to be surface exposed although it does appear, nevertheless, to elicit the production of bactericidal antibodies. As with RmpM and PilQ, it is possible that alternative structural states of these complex assemblies exist *in vivo*, perhaps during biogenesis. It is interesting to note that we observed several correlations in antibody responses for BamA and BamC against other proteins and it is tempting to speculate that these correlations reflect the central role that the BAM complex plays in outer membrane insertion. There is at present, however, no direct evidence to support this idea.

We noted a significant stimulation of IgG antibodies against the lipoprotein GNA1162 (NEISS1063) in our previous study [18]. We also observed that this antigen has limited sequence variability. Here, it is striking that correlations were also detected against log(SBA) in three out of the five strains studied (Tables 1 and 2). Studies on this protein as a vaccine component have been limited; it was identified in the reverse vaccinology screen by Pizza *et al*. but not further developed [9]. Our observations suggest that it may be worth re-visiting as a vaccine component.

The antigen MtrE was correlated with SBA titers in two of the five meningococci analysed, 3208 and 3043, which both contained different PorA variants from the wild type. It may be that MtrE has a higher level of expression in these organisms. MtrE is part of an efflux pump complex and mediates resistance to antibiotics. The crystal structure of the protein from *N. gonorrhoeae*, which is highly homologous to MtrE from *N. meningitidis*, showed a similar structure to *E. coli* TolC [50]. Each MtrE chain comprises 4 trans-membrane β-strands which assemble, as a trimer, into a 12-stranded β-barrel with extensive α-helical domains which span the periplasm and provide a channel for passage of the transported substrate. Just two loops from the barrel are predicted to be surface exposed; however, MtrE from *N. gonorrhoeae* has been shown to elicit bactericidal antibodies in mice [51] and there is recent evidence that 4CMenB induces antibodies which recognise MtrE from several gonococci [52]. There is therefore some evidence to support the idea MtrE is a minor antigen in OMV preparations.

The examination of PorA- meningococci facilitated the identification of minor antigens that are potentially correlated with SBA responses. One explanation for this observation is that these meningococci are targeted by a higher proportion of antibodies against minor antigens, i.e. deletion of PorA can lead to an increase in the relative abundance of other OMPs. This included, in one instance, the 4CMenB component fHbp [11]. We would therefore advocate the use of a diverse range of variant meningcocci for SBA analyses which could identify further potential vaccine candidates. The other factor influencing the sensitivity with which antigens are detected is likely to be the number of serum samples: a greater range and diversity of responses is likely to improve the sensitivity with which correlations are detected. It would also be useful to repeat the study using sera from infants.

It is arguable that development of a complex, multicomponent vaccine requires a consideration of the way in which individual antigens induce immunogenic responses within the context of the complete, formulated product. For example, non-protein components, such as LPS, can have adjuvant properties that lead to improved immunogenic responses. Further, it can be argued that use of antibody responses from an entire OMV also replicates more closely natural infection, whereby multiple antigens are presented simultaneously to antigen presenting cells. An advantage of a more holistic approach to antigen identification is that it does not involve the testing of antigens one by one, which can be a tedious, low throughput process. Finally, mouse immune responses may not be an infallible guide to those found in humans; indeed, we observed differences in our previous study [18]. Overall, this suggests that engineering the content of OMVs to optimize the expression of antigens that correlate well with SBA titres could be a useful strategy in the future.

## Supplementary Material

**Appendix A. Supplementary data**

Supplementary data to this article can be found online at https://doi.org/10.1016/j.vaccine.2022.05.032.

Suplp2

Suppl1

## Figures and Tables

**Table 1:** Kendall’s tau statistical correlations between SBA and IgG responses to antigens.

Antigen	Wild Type			PorA- FetA+			PorA- FetA-			3208 FetA+			3043 FetA-		
	tau	Sigma^1^		tau	Sigma		tau	Sigma		tau	Sigma		tau	Sigma	
PorA	0.53	5.9E-10		0.31	3.0E-04					0.52	6.0E-10		0.55	5.5E-11	
RmpM	0.37	1.6E-05		0.32	2.3E-04		0.24	7.8E-03		0.42	5.7E-07		0.40	2.1E-06	
OpcA (multimer)	0.31	2.8E-04								0.39	4.8E-06		0.40	1.9E-06	
PorB	0.34	8.3E-05		0.30	4.5E-04					0.35	3.3E-05		0.30	4.5E-04	
OpcA (monomer)	0.33	1.2E-04								0.40	2.3E-06		0.42	6.1E-07	
PilQ (25–777)	0.26	2.5E-03		0.38	9.9E-06					0.29	5.7E-04		0.25	3.8E-03	
BamA	0.27	2.1E-03								0.30	3.8E-04		0.30	4.1E-04	
Lipoprotein (GNA1162)^2^	0.25	3.7E-03					0.28	1.6E-03					0.28	9.8E-04	
BamC				0.26	3.9E-03					0.23	7.6E-03				
DSBC thiosulfide				0.25	3.8E-03		0.24	5.9E-03							
PilQ (25–580)				0.32	2.5E-04										
Putative uncharacterized protein^3^				0.30	6.6E-04										
Peptidyl-prolyl cis–trans isomerase							0.23	8.5E-03							
Factor H binding protein							0.27	2.8E-03							
Aminodeoxychorismate lyase family protein							0.25	5.7E-03							
MtrE										0.24	7.5E-03		0.23	7.9E-03	
Gamma glutamyltransferase													0.23	7.0E-03	

1 Two-tailed; only correlations with p < 0.01 are included; values were not normalized before analysis.

2 UNIPROT code E6MWK5.

3 UNIPROT code E6MZZ3.

**Table 2:** Spearman’s correlation coefficient statistical correlations between SBA and IgG responses to antigens.

Antigen	Wild Type			PorA- FetA+			PorA- FetA-			3208 FetA+			3043 FetA-		
	c.c.^1^	Sigma^2^		c.c.	Sigma		c.c.	Sigma		c.c.	Sigma		c.c.	Sigma	
PorA	0.70	8.5E-12		0.42	1.9E-04					0.70	5.7E-12		0.74	9.5E-14	
RmpM	0.52	4.1E-06		0.43	1.1E-04		0.32	5.4E-03		0.58	6.6E-08		0.55	3.7E-07	
OpcA (multimer)	0.44	1.5E-04								0.53	1.3E-06		0.55	3.8E-07	
PorB	0.46	5.1E-05		0.40	3.5E-04					0.47	2.0E-05		0.40	3.9E-04	
OpcA (monomer)	0.49	1.7E-05								0.56	1.8E-07		0.59	3.0E-08	
PilQ (25–777)	0.37	1.4E-03		0.52	2.5E-06					0.41	2.7E-04		0.36	1.8E-03	
BamA	0.36	1.9E-03								0.41	3.0E-04		0.41	3.2E-04	
Lipoprotein (GNA1162)^3^	0.34	3.4E-03					0.38	7.9E-04					0.37	1.0E-03	
BamC				0.33	3.7E-03					0.32	5.3E-03				
DSBC thiosulfide				0.33	3.6E-03		0.32	6.0E-03							
PilQ (25–580)				0.43	1.5E-04										
Putative uncharacterized protein^4^				0.39	6.3E-04										
Peptidyl-prolyl cis–trans isomerase							0.31	6.6E-03							
LysM domain							0.30	8.4E-03							
Aminodeoxychorismate lyase family protein							0.34	3.4E-03							
Factor H binding protein							0.36	1.6E-03							
MtrE										0.33	4.7E-03		0.32	5.5E-03	
Gamma glutamyltransferase													0.33	3.9E-03	

1 Correlation coefficient (rho); only correlations with p < 0.01 are included; values were not normalized before analysis.

2 Two-tailed.

3 UNIPROT code E6MWK5.

4 UNIPROT code E6MZZ3.

**Table 3:** ANOVA comparison of linear regression fitting models.^1^

	Wild Type	3208 FetA+	3043 FetA-
F-statistic	3.67	2.55	2.71
p-value	0.00342	0.0277	0.0206

1 ANOVA comparison between linear regression models fitted either to PorA alone (model 1), or PorA + RmpM + OpcA(multimer) + PilQ(25–777) + BamA + lipoprotein GNA1162 (model 2).
