# Supplementary material for: Application of a Neisseria meningitidis antigen microarray to identify candidate vaccine proteins from a human Phase I clinical trial
Source: Vaccine. Author manuscript; Available in PMC 2024 Sep 24. (PMC7616631; doi:10.1016/j.vaccine.2022.05.032)
Supplement: Suplp2 [file EMS198675-supplement-Suplp2.docx]

| antigen1 |  | antigen2 |  | F-statistic |
| --- | --- | --- | --- | --- |
| Transglycosylase (dimer) | NEIS1920 | Transglycosylase (monomer) | NEIS1920 | 1926 |
| Peptide_methionine_sulfoxide_reductase (trimer/tetramer) | NEIS0020 | Peptide_methionine_sulfoxide_reductase (octamer) | NEIS0020 | 887.4 |
| putrescine-binding_protein (dimer) | NEIS1689 | putrescine-binding_protein (monomer) | NEIS1689 | 731.9 |
| periplasmic_cystine_binding_protein (dimer) | NEIS0739 | periplasmic_cystine_binding_protein (monomer) | NEIS0739 | 706.2 |
| periplasmic_putrescine-binding_protein (monomer) | NEIS0567 | periplasmic_putrescine-binding_protein (dimer) | NEIS0567 | 606.3 |
| Ahpc_TSA_antioxidant | NEIS1498 | Phospholipase_Patain_Family | NEIS2118 | 583.4 |
| OpcA (multimer) | NEIS2198 | OpcA (monomer) | NEIS2198 | 558.5 |
| FetA (multimer) | NEIS1963 | FetA (monomer) | NEIS1963 | 253.7 |
| NspA (multimer) | NEIS0612 | NspA (monomer) | NEIS0612 | 196.9 |
| YceI_family_protein (multimer) | NEIS1183 | YceI_family_protein (monomer) | NEIS1183 | 156.1 |
| PilQ_25-777 | NEIS0408 | BamC | NEIS0906 | 124.7 |
| PotF1 (monomer) | NEIS1516 | PotF1 (tetramer) | NEIS1516 | 121.1 |
| PilQ_25-777 | NEIS0408 | PilQ 25-580 | NEIS0408 | 84.3 |
| Heme-utilization_protein_Hup | NEIS1428 | OmpH | NEIS0172 | 83.1 |
| BamE | NEIS0196 | fimbrial_protein (PilE) | NEIS0210 | 80.1 |
| DSBA_Thioredoxin_domain_Protein | NEIS0273 | periplasmic_putrescine-binding_protein (dimer) | NEIS0567 | 78.5 |
| TbpB | NEIS1691 | PotF1 (tetramer) | NEIS1516 | 78.2 |
| putrescine-binding_protein (monomer) | NEIS1689 | Ahpc_TSA_antioxidant | NEIS1498 | 66.3 |
| DSBA_Thioredoxin_domain_Protein | NEIS0273 | periplasmic_putrescine-binding_protein (monomer) | NEIS0567 | 55.9 |
| putrescine-binding_protein (monomer) | NEIS1689 | Phospholipase_Patain_Family | NEIS2118 | 54.9 |
| MafA (decamer) | NEIS2083 | periplasmic_putrescine-binding_protein (dimer) | NEIS0567 | 51.9 |
| DSBA_thioredoxin_domain_protein | NEIS1760 | Peptide_methionine_sulfoxide_reductase | NEIS0020 | 51.5 |
| putrescine-binding_protein (dimer) | NEIS1689 | Ahpc_TSA_antioxidant | NEIS1498 | 48.6 |
| TbpB | NEIS1691 | PotF1 (monomer) | NEIS1516 | 48.6 |
| Transglycosylase | NEIS1920 | Transglycosylase_SLT_domain_protein | NEIS1920 | 48.1 |
| Lipoprotein_Mlp | NEIS0325 | Ahpc_TSA_antioxidant | NEIS1498 | 46.6 |
| MIP | NEIS1487 | Transglycosylase | NEIS1920 | 46 |
| Homoserine_dehydrogenase | NEIS1128 | Heme-utilization_protein_Hup | NEIS1428 | 44.7 |
| putrescine-binding_protein (dimer) | NEIS1689 | Phospholipase_Patain_Family | NEIS2118 | 42.9 |
| Peptide_methionine_sulfoxide_reductase | NEIS0020 | DSBA_thioredoxin_domain_protein | NEIS1760 | 42.7 |
| Lipoprotein_Mlp | NEIS0325 | Phospholipase_Patain_Family | NEIS2118 | 41.7 |
| Lipoprotein | NEIS0071 | MtrE (trimer) | NEIS1632 | 39.9 |
| MafA (decamer) | NEIS2083 | periplasmic_putrescine-binding_protein (monomer) | NEIS0567 | 39 |
| BamE | NEIS0196 | FetA (monomer) | NEIS1963 | 36.8 |
| DSBA_thioredoxin_domain_protein | NEIS1760 | Hemolysin_Secretion_Activation_ShlB | NEIS2501 | 36.5 |
| LysM_domain | NEIS0101 | YceI_family_protein | NEIS1183 | 35.8 |
| MIP | NEIS1487 | Transglycosylase | NEIS1920 | 35.7 |
| Transglycosylase | NEIS1920 | Transglycosylase_SLT_domain_protein | NEIS1920 | 35.3 |
| PPIC-type_PPIASE | NEIS0276 | LolA | NEIS0566 | 34.5 |
| periplasmic_substrate_binding_protein | NEIS0528 | Uncharacterized_Protein | NEIS0735 | 33.9 |
| NspA (multimer) | NEIS0612 | Enoyl_acyl_carrier_protein_reductase | NEIS1834 | 33.6 |
| Lipoprotein | NEIS1917 | Conserved_Hypothetical_Protein | NEIS1824 | 32.7 |
| Transglycosylase_SLT_domain_protein | NEIS1920 | Superoxide_dismutase | NEIS1339 | 31.9 |
| conserved_hypothetical_protein | NEIS1934 | Hypothetical_protein | NEIS1825 | 31.5 |
| DSBA_Thioredoxin_domain_Protein | NEIS0273 | periplasmic_iron_binding_protein | NEIS0578 | 31.5 |
| FetA (monomer) | NEIS1963 | fimbrial_protein (PilE) | NEIS0210 | 31.5 |

**Table S1 Top 46 highest correlated antigen pairs by F-statistic.**
