# Supplementary material for: Application of a Neisseria meningitidis antigen microarray to identify candidate vaccine proteins from a human Phase I clinical trial
Source: Vaccine. Author manuscript; Available in PMC 2024 Sep 24. (PMC7616631; doi:10.1016/j.vaccine.2022.05.032)
Supplement: Suppl1 [file EMS198675-supplement-Suppl1.docx]

**Supplementary Figures**


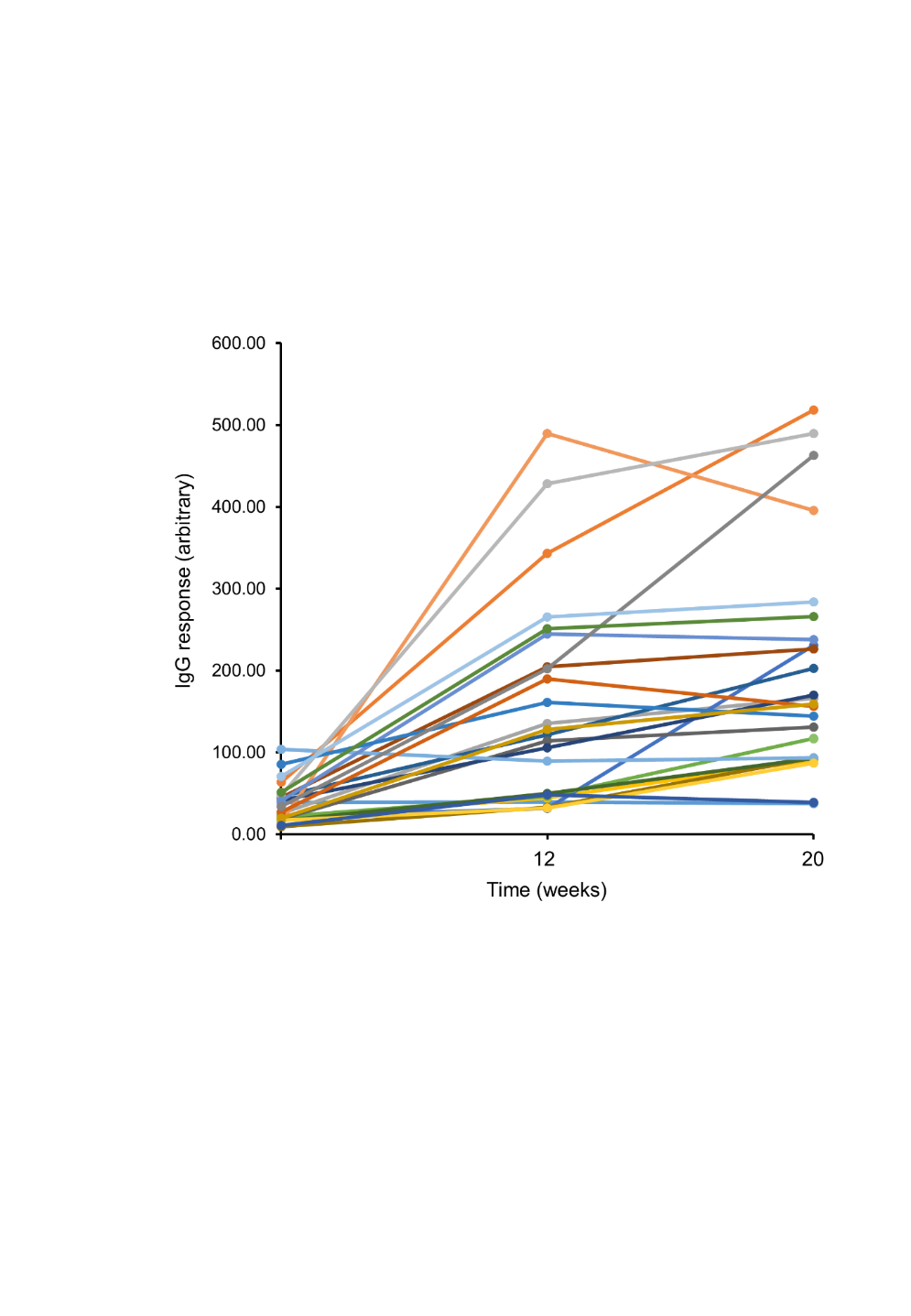


**Figure S1. Variation in IgG responses against PorA antigen following MenPF vaccination.**


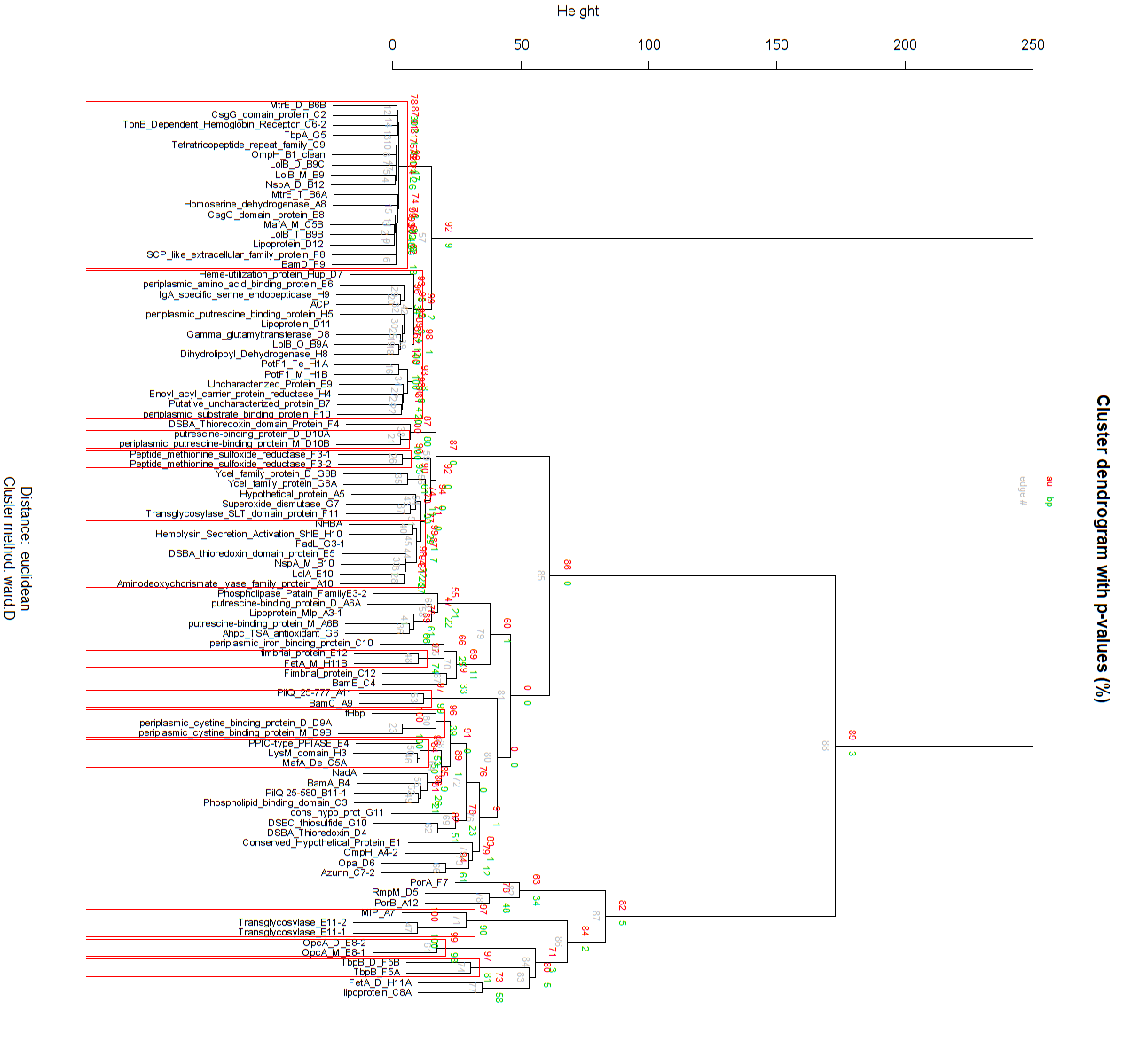


**Figure S2. Hierarchical clustering of IgG antigen responses.** AU is Approximately Unbiased p-value (%) and BP Bootstrap Probability value (%). Clustering (Ward) was carried out using pvclust (Suzuki, R. & Shimodaira, H. Pvclust: an R package for assessing the uncertainty in hierarchical clustering. Bioinformatics 22, 1540-1542, doi:10.1093/bioinformatics/btl117 (2006).) Clustering was carried out after normalization of the data with caret. Clusters with p-values less than 0.05 are highlighted in red.
